# Effects of turbinoplasty versus outfracture and bipolar cautery on the compensatory inferior turbinate hypertrophy in septoplasty patients^☆^

**DOI:** 10.1016/j.bjorl.2018.04.010

**Published:** 2018-05-18

**Authors:** Aykut Bozan, Hüseyin Naim Eriş, Denizhan Dizdar, Sercan Göde, Bahar Taşdelen, Hayrettin Cengiz Alpay

**Affiliations:** a Istanbul Kemerburgaz University, Medical Faculty, Department of Otorhinolaryngology, Tarsus, Turkey; b Medical Park Tarsus Hospital, Radiology, Tarsus, Turkey; c Ege University, Medical Faculty, Department of Otorhinolaryngology, İzmir, Turkey; d Mersin University, Medical Faculty, Biostatistics, Mersin, Turkey

**Keywords:** Turbinate, Hypertrophy, Outfracture, Turbinoplasty, Concha, Hipertrofia, Fratura lateral, Turbinoplastia

## Abstract

**Introduction:**

The most common cause of septoplasty failure is inferior turbinate hypertrophy that is not treated properly. Several techniques have been described to date: total or partial turbinectomy, submucosal resection (surgical or with a microdebrider), with turbinate outfracture being some of those.

**Objective:**

In this study, we compared the pre- and postoperative lower turbinate volumes using computed tomography in patients who had undergone septoplasty and compensatory lower turbinate turbinoplasty with those treated with outfracture and bipolar cauterization.

**Methods:**

This retrospective study enrolled 66 patients (37 men, 29 women) who were admitted to our otorhinolaryngology clinic between 2010 and 2017 because of nasal obstruction and who were operated on for nasal septum deviation. The patients who underwent turbinoplasty due to compensatory lower turbinate hypertrophy were the turbinoplasty group; Outfracture and bipolar cauterization were separated as the out fracture group. Compensatory lower turbinate volumes of all patients participating in the study (mean age 34.0 ± 12.4 years, range 17–61 years) were assessed by preoperative and postoperative 2 month coronal and axial plane paranasal computed tomography.

**Results:**

The transverse and longitudinal dimensions of the postoperative turbinoplasty group were significantly lower than those of the out-fracture group (*p* = 0.004). In both groups the lower turbinate volumes were significantly decreased (*p* = 0.002, *p* < 0.001 in order). The postoperative volume of the turbinate on the deviated side of the patients was significantly increased: tubinoplasty group (*p* = 0.033).

**Conclusion:**

Both turbinoplasty and outfracture are effective volume-reduction techniques. However, the turbinoplasty method results in more reduction of the lower turbinate volume than outfracture and bipolar cauterization.

## Resumo

**Introdução:**

A causa mais comum de falha da septoplastia é a hipertrofia das conchas inferiores não tratada adequadamente. Diversas técnicas foram descritas até o momento: turbinectomia total ou parcial, ressecção da submucosa (cirúrgica ou com microdebridador) e a fratura lateral.

**Objetivo:**

Neste estudo, comparamos os volumes pré e pós-operatório da concha inferior com hipertrofia compensatória com o uso de tomografia computadorizada entre pacientes submetidos a septoplastia e turbinoplastia ou fratura lateral com cauterização bipolar.

**Método:**

Este estudo retrospectivo incluiu 66 pacientes (37 homens e 29 mulheres) internados em nosso serviço de otorrinolaringologia entre 2010 e 2017 por obstrução nasal e submetidos à cirurgia por desvio de septo nasal. Os pacientes submetidos à turbinoplastia devido à hiperplasia compensatória da concha inferior formaram o grupo turbinoplastia; aqueles submetidos à fratura lateral e cauterização bipolar foram separados, formaram o grupo fratura lateral. Os volumes compensatórios da concha inferior de todos os pacientes que participaram do estudo (idade média de 34,0 ± 12,4 anos, faixa de 17 a 61 anos) foram avaliados por tomografia computadorizada dos seios paranasais nos planos axial e coronal no pré-operatório e aos dois meses do pós-operatório.

**Resultados:**

As dimensões transversais e longitudinais do grupo turbinoplastia no pós-operatório foram significantemente menores do que as do grupo de fratura lateral (*p* = 0,004). Em ambos os grupos, os volumes da concha inferior diminuíram significantemente (*p* = 0,002, p < 0,001, respectivamente). O volume pós-operatório da concha do lado do desvio aumentou significantemente no grupo turbinoplastia (*p* = 0,033).

**Conclusão:**

Tanto a turbinoplastia como a fratura lateral são técnicas efetivas de redução de volume. No entanto, a turbinoplastia causa maior redução do volume da concha inferior do que a fratura lateral com cauterização bipolar.

## Introduction

The most common cause of chronic nasal obstruction is septum deviation and lower turbinate pathologies.^1^ Inferior turbinate hypertrophy is frequently seen in allergic rhinitis, vasomotor rhinitis, and as compensatory hypertrophy in septum deviation. Lower turbinate hypertrophy on the concave side of the nasal septum is called compensatory hypertrophy.^2^ The most common cause of septoplasty failure is inferior turbinate hypertrophy that is not treated properly.^3^ Several techniques have been described to date: total or partial turbinectomy, submucosal resection (surgical or with a microdebrider), outfracture, electrocautery, radiofrequency application, argon plasma treatment, and cryosurgery.^4^

None of the turbinate surgical techniques performed with or without septoplasty are perfect. Short- and long-term complications, such as bleeding, bruising, and atrophy, are frequent.^5^ Ideally, turbinate surgery should be done without damaging the mucosal surface. This ensures preservation of normal lower turbinate function, rapid healing, and inhibition of atrophic rhinitis.^6^ Despite the increasing number of lower turbinate surgical procedures, turbinoplasty, outfracture, and bipolar cautery methods have been used frequently for the last three decades.^7^ Turbinoplasty is more difficult and has a higher complication rate than the outfracture method, despite its high success rate. Lower turbinate outfracture and bipolar cauterization can be applied in the same order and more quickly.^8^

In this study, we compared the pre- and postoperative lower turbinate volumes using computed tomography (CT) in patients who had undergone septoplasty and compensatory lower turbinate turbinoplasty with those treated with outfracture and bipolar cauterization.

## Methods

### Patient selection

This retrospective study enrolled 66 patients (37 men, 29 women) who were admitted to our otorhinolaryngology clinic between 2010 and 2017 because of nasal obstruction and who were operated on for nasal septum deviation. CT showed septum deviation and contralateral compensatory lower turbinate hypertrophy. The patients were divided into two groups. The turbinoplasty group included patients who underwent septoplasty and turbinoplasty; the outfracture group underwent septoplasty with compensatory lower turbinate outfracture and bipolar cauterization.

Patients with maxillofacial trauma, paranasal sinus tumors, nasal polyps, septal perforations, acute or chronic rhinosinusitis, S type nasal septum deviation, turbinate bullosa, or previous nasal or paranasal surgery were excluded from the study. Ethics committee approval was obtained from Istanbul University, Cerrahpaşa Medical Faculty, Ethical Committee (n° 61328).

### Surgical procedure

All patients were operated by the same surgeon under general anesthesia. First, a septoplasty was performed. Thirty-two patients (19 men, 13 women; mean age, 36.6 ± 15.0 years, range: 19–61 years) in the turbinoplasty group underwent compensatory lower turbinate turbinoplasty. A superior-to-inferior incision was made on the anterior surface of the lower turbinate with a n° 15 blade, working under a 0° endoscopic video image, and this incision was extended posteriorly along the inferior surface. The medial side of turbinate was elevated. The turbinatel mucosa and turbinate were excised while preserving the medial flap. Bleeding was controlled with bipolar cauterization. The flap was replaced, packing was placed in both nasal cavities, and the operation completed. Nasal packing was removed after 48 h.

The outfracture group comprised 44 patients (18 men, 16 women; mean age, 31.4 ± 9.5 years, range: 17–49 years) who underwent turbinate outfracture and bipolar cauterization. Using an elevator, the lower turbinate was first mobilized medially and laterally. Posterior anterior bipolar cauterization was then applied to the inferomedial face of the lower turbinate. Both nasal cavities were filled with nasal cuffs and the operation completed. Nasal packing was removed after 48 h.

### Patient evaluation

The compensatory turbinatel volume of all subjects was assessed pre- and postoperatively using coronal and axial plane paranasal CT performed in 1 mm sections from anterior (nares) to posterior (choana). The volumetric evaluations were performed by the same radiologist.

The lower turbinate volumes were calculated in mm^3^ using the ellipse formula: longitudinal dimension (mm) × transverse dimension (mm) × anteroposterior dimension (mm) × 0.52. The longitudinal and transverse turbinate dimensions were calculated from the cross-section through the coronal plane after the uncinate processes. The longest dimension of the lower turbinate was set as the anteroposterior dimension in the axial plane.

### Statistical analysis

Statistical analysis was performed using STATA/MP 11. The data were summarized as means and standard deviation. Pre- and postoperative comparisons were made using paired *t*-tests within each group. The independent *t*-test was used to compare preoperative groups, while analysis of covariance (ANCOVA) was used to compare postoperative groups using the preoperative values as covariates. The independent *t*-test was used to compare relative postoperative changes (%) between groups. Statistical significance was taken as *p* < 0.05.

## Results

Endoscopic hemorrhage control was performed because of hemorrhage development on postoperative 4th and 6th days in postoperative period in only 2 patients in the group of turbinoplasty. In the other 64 patients, there were no complications such as postoperative hemorrhage, synechia or infection. Nasal endoscopic examinations were performed at 2 months postoperatively. No signs of septum deviation, turbinate hypertrophy, or atrophic rhinitis were observed in the follow-up examinations, and there were no complaints of nasal obstruction.

The differences in the pre- and postoperative parameters were significant in the turbinoplasty and outfracture groups (Table 1).

**Table 1:** Compensatory turbinate preoperative and postoperative values.

	Turbinoplasty			Out fracture		
	Preop.	Postop.	*p*	Preop.	Postop.	*p*
A-P (mm)	48.1 ± 4.8	39.4 ± 4.9	**0.009**	43.7 ± 6.6	38.2 ± 6.6	**0.001**
Transverse (mm)	11.4 ± 2.2	6.2 ± 1.5	**<0.001**	12.1 ± 2.2	9.6 ± 2.7	**<0.001**
Longitudinal (mm)	17.8 ± 2.9	11.5 ± 2.5	**<0.001**	14.9 ± 2.8	12.8 ± 2.6	**0.005**
Volume (mm^3^)	4523.5 ± 1548.2	1492.2 ± 594.8	**0.002**	4282.6 ± 2094.2	2699.9 ± 1942.1	**<0.001**

Mean and standard deviation were defined for each subgroup. Statistically significant results are shown in bold.

A-P, Anterior-Posterior; Preop preoperative, Postop postopertative; mm, milimeter.

The transverse and longitudinal dimensions of the lower turbinate in the turbinoplasty group were significantly lower than in the outfracture group (*p* = 0.004). The postoperative lower turbinate volumes decreased significantly in both the turbinoplasty and outfracture groups. In the turbinoplasty group, the mean lower turbinate volume was 4523.5 mm^3^ preoperatively and 1492.2 mm^3^ postoperatively (*p* = 0.002), versus 4282.2 mm^3^ preoperatively and 2699.9 mm^3^ postoperatively (*p* < 0.001) in the outfracture group. Comparing the turbinoplasty and outfracture groups, the postoperative volume was significantly lower in the turbinoplasty group (*p* = 0.019) (Table 2). In the between-group comparison, the volume reduction was greater in the turbinoplasty group (*p* = 0.037) (Table 2).

**Table 2:** Changes in turbinate measures.

	Turbinoplasty	Out fracture	*p*
Preop A-P (mm)	48.1 ± 4.8	43.7 ± 6.6	0.188
Postop A-P (mm)	39.4 ± 4.9	38.2 ± 6.6	0.490
Decrease longitudinal (mm)	0.17 ± 0.11	0.13 ± 0.05	0.336
Preop transvers (mm)	11.4 ± 2.2	12.1 ± 2.2	0.576
Postop transvers (mm)	6.2 ± 1.5	9.6 ± 2.7	**0.004**
Decrease transvers (mm)	0.45 ± 0.12	0.22 ± 0.08	**0.001**
Preop longitudinal (mm)	17.8 ± 2.9	14.9 ± 2.8	0.08
Postop longitudinal (mm)	11.5 ± 2.5	12.8 ± 2.6	**0.004**
Decrease longitudinal (mm)	0.36 ± 0.09	0.14 ± 0.08	**<0.001**
Preop volüm (mm^3^)	4523.5 ± 1548.2	4282.6 ± 2094.2	0.811
Postop volüm (mm^3^)	1492.2 ± 594.8	2699.9 ± 1942.1	**0.019**
Decrease volüm (mm^3^)	0.63 ± 0.34	0.41 ± 0.12	**0.037**

Mean and standard deviation were defined for each subgroup. Statistically significant results are shown in bold.

A-P, Anterior-Posterior; Preop preoperative, Postop postopertative; mm, milimeter.

The transverse and longitudinal dimensions of the lower turbinate decreased more in the turbinoplasty group compared with the outfracture group (*p* = 0.001 and *p* < 0.001, respectively) (Table 2).

**Figure 1:**
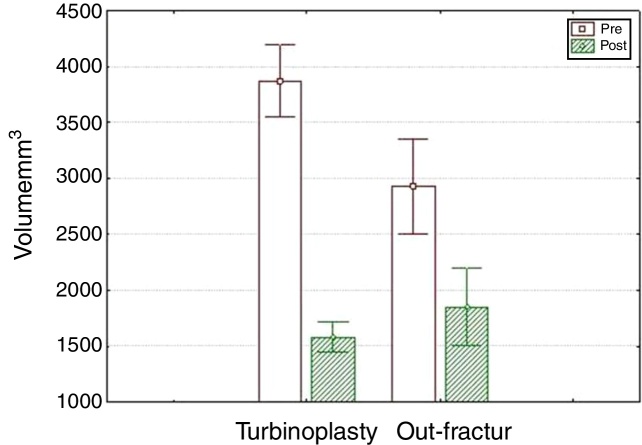
Preoperative and postoperative volume changes of the groups.

In the turbinoplasty group, the turbinate volume had an average reduction of 56% and 36% in the out-fracture group (Fig. 1).

The lower turbinate volumes on the side of the deviation were significantly increased in both the turbinate and out-fracture groups postoperatively (*p* = 0.0002, *p* = 0.0297, respectively) (Table 3).

**Table 3:** Lower turbinate volumes on deviated side.

	Turbinoplasty			Out fracture		
	Preop.	Postop.	*p*	Preop.	Postop.	*p*
Volume (mm^3^)	1967.8 ± 426.1	2070. ± 413.8	<**0.0002**	1725.2 ± 327.2	1791.1 ± 340.3	<**0.0297**

Statistically significant results are shown in bold.

## Discussion

A compensatory turbinate develops to protect the more-involved nasal passage from cold, dry air. The most common site is the inferior turbinate. There is thickening of the turbinate bones, and an increase in the spongiform structure and orientation to the midline. Mucosal hypertrophy is also present.^9^ Many techniques have been described to reduce the volume in lower turbinate hypertrophy. In some of these techniques, the aim is only to decrease the mucosal volume, while in others the mucous membrane and bone volume are both reduced.^10^ There is no consensus regarding the best lower turbinate reduction technique. Although less invasive methods have become popular over the last 20 years, more invasive procedures, such as turbinoplasty, remain important because of their high success rates.

Many studies have examined the effectiveness of radiofrequency application in lower turbinate surgery,^10^, ^11^, ^12^ and other techniques have been evaluated in non-septoplasty patients.^13^, ^14^, ^15^ Veit et al. did not evaluate lower turbinate volumes despite comparing lower turbinate reduction methods during septoplasty.^16^

We measured the turbinate volume using CT and compared the volume after outfracture and bipolar cauterization, which caused only mucosal volume loss, with that of turbinoplasty, which resulted in mucosal and bone volume loss during septoplasty. Other studies have measured the volume using CT (10) or magnetic resonance imaging.^13^, ^17^

Turbinoplasty is a successful method despite postoperative synechia, drying, and nasal discharge problems.^16^, ^18^ In our study, postoperative desiccation and nasal discharge was not followed up in the turbinoplasty patients.

Büyüklü and Zhang^19^, ^20^ reported that the outfracture method was effective for expanding the nasal passages in lower turbinate hypertrophy. With turbinate bipolar cauterization, superficial thermal ablation creates scar tissue and fibrosis, and obliterates the venous sinuses. In one study, the results at 2 months after bipolar cauterization were successful in 76% of the cases.^14^ In our study, the lower turbinate volume in the outfracture group decreased significantly and the patients’ complaints of nasal obstruction disappeared. In both groups, the improvement in the nasal obstruction was likely related to both the lower turbinate reduction and correction of the septum deviation.

Various studies have compared the effectiveness of lower turbinate surgical techniques using objective tests such as acoustic rhinomanometry, mucociliary function tests, and acoustic rhinometry.^15^, ^21^, ^22^

Can et al.^13^ have studied the effects of radiofrequency ablation in patients undergoing lower turbinate submucosal resection and found that the volume reduction was significant in both groups, but it was greater with radiofrequency ablation. In our study, the postoperative axial, transverse, and longitudinal lower turbinate dimensions were decreased significantly in both groups.

Changes in lower turbinate volume have been assessed after applying different reduction methods. Demir et al.^12^ found that the lower turbinate volume decreased by 25% after thermal radiofrequency ablation. Can et al.^13^ reported a 42.4% volume reduction after submucosal resection. We observed greater volume reduction in the turbinoplasty group (67.1%) than the outfracture group (36.9%), indicating that hypertrophic mucosa and bone formation with compensatory hypertrophy constitutes a significant volume. Furthermore, the decrease in the transverse and longitudinal dimensions of the lower turbinate was significantly (*p* < 0.001) greater in our turbinoplasty group compared with the outfracture group, and the loss in the turbinoplasty group could be attributed to bone tissue loss. Turbinoplasty method results in a greater volume decrease and can be selected for lower turbinate in which the bone mass produces a significant volume, while outfracture and bipolar cauterization, which has a lower risk of complications, can be performed in patients with more moderate lower turbinate hypertrophy.

Lower turbinate outfracture and bipolar cauterization are less invasive than turbinoplasty, while the risk of perioperative bleeding is greater than with turbinoplasty.^18^ While hemorrhage, synechiae, and mucosal discharge can occur after turbinoplasty, these effects are not observed after outfracture and bipolar cauterization. In addition, turbinoplasty is suitable for bleeding control under an endoscopic view. Consequently, turbinoplasty takes longer to perform than outfracture and bipolar cauterization. In our series, no peri- or postoperative complications were recorded in either group, but this may be due to the small number of subjects.

In a comparison of the pre- and postoperative lower turbinate volumes of patients who underwent radiofrequency ablation of the lower turbinate, Bahadır et al.^10^ stated that the postoperative volumes of six lower turbinate were increased, which might have been due to the stage of the nasal cycle. In our study, the significant increase in the volume of the uninvolved lower turbinate (*p* = 0.033) on the deviated side in the turbinoplasty group might have been due to a process other than the nasal cycle following correction of the deviation.

## Conclusion

Both turbinoplasty and outfracture are effective volume reduction techniques. However, the turbinoplasty method causes more reduction of the lower turbinate volume of the than outfracture and bipolar cauterization

## Ethical approval

All procedures performed in studies involving human participants were in accordance with the ethical standards of the institutional and/or national research committee and with the 1964 Helsinki declaration and its later amendments or comparable ethical standards.

## Informed consent

Informed consent was obtained from all individual participants included in the study.

The English in this document has been checked by at least two professional editors, both native speakers of English. For a certificate, please see: http://www.textcheck.com/certificate/eqNE75.

## Conflicts of interest

The authors declare no conflicts of interest.
